# Synthesis and Encapsulation of *Ajuga parviflora* Extract with Zeolitic Imidazolate Framework-8 and Their Therapeutic Action against G^+^ and G^–^ Drug-Resistant Bacteria

**DOI:** 10.1021/acsomega.1c03984

**Published:** 2022-01-06

**Authors:** Ab Majeed Ahanger, Suresh Kumar, Atul Arya, Amrita Suryavanshi, Dolly Kain, Vandana

**Affiliations:** † Medicinal Plant Research Laboratory, Department of Botany, Ramjas College, University of Delhi, New Delhi 110007, India; ‡ Department of Chemistry, Dyal Singh College, University of Delhi, New Delhi 110003, India

## Abstract

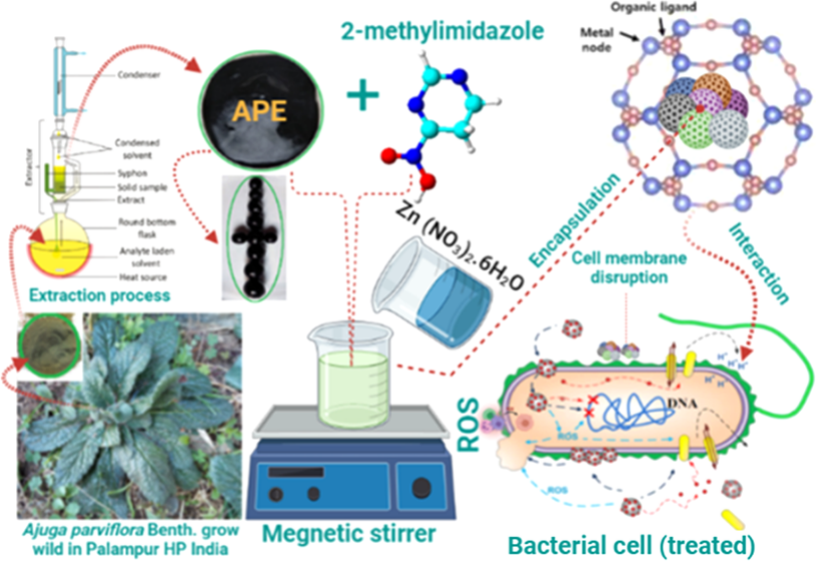

Infectious diseases caused by bacteria have become a public health issue. Antibiotic therapy for infectious disorders, as well as antibiotic overuse, has resulted in antibiotic-resistant bacterial strains. Zeolitic imidazolate framework-8 (ZIF-8) possesses a wide surface area, high porosity, variable functionality, and potential drug carriers. We have established a clear method for making a nanoscale APE@ZIF-8 nanocomposite agent with outstanding antibacterial activity against methicillin-resistant *Staphylococcus aureus* (MRSA) and cephalosporin-carbapenem-resistant *Escherichia coli* (CCREC). We present a unique approach for encapsulating molecules of*Ajuga parviflora* extract (APE) with ZIF-8. APE@ZIF-8 has a positive charge. By electrostatic contact with the negatively charged bacterial surface of *S. aureus* and *E. coli*, APE@ZIF-8 NPs produce reactive oxygen species (ROS) that damage bacterial cell organelles. As a result, the APE@ZIF-8 nanocomposite offers limitless application potential in the treatment of infectious disorders caused by drug-resistant gram-positive and gram-negative bacteria.

## Introduction

Since the emergence of antibiotic resistance, bacterial-mediated infectious diseases have become a major health concern.^1^ Antibiotic resistance occurs when bacteria develop their ability to withstand medications that kill them, allowing them to grow and become useless. Antimicrobial resistance is a global concern for human health and development. The World Health Organization (WHO) has issued a report calling for fast, coordinated, and ambitious action to avoid a devastating drug-resistance disaster. Drug-resistant diseases could kill 10 million people per year by 2050 if nothing is done. Drug-resistant infections claim the lives of at least 700,000 people each year, and nations spend heavily on innovative research and technology to tackle antibiotic resistance.^2^ Methicillin-resistant *Staphylococcus aureus* (MRSA) is a gram-positive bacteria that causes blood poisoning (bacteremia),^3^ toxic shock syndrome, renal failure, and pneumonia in people all over the world.^4^ According to the Centers for Disease Control and Prevention, *E. coli* is resistant to cephalosporins (Cefotaxime).^5^ A mutant strain of *Escherichia coli* is resistant to carbapenem (imipenem), according to a paper published by the Indian Council of Medical Research in 2020.^6^ UTIs, renal failure, and newborn meningitis are all caused by multidrug-resistant *E. coli* virulence genes.^7−10^ Antibacterial agents can effectively penetrate gram-positive bacteria’s thick but porous cell walls, which contain peptidoglycan (20–80 nm in size) made up of N-acetylated muramic acid, glucosamine, and teichoic acid and bear a strong negative charge, but gram-negative bacteria have bilayer membranes with 5–10 nm outer membranes of negatively charged oligosaccharides and lipoproteins, and as a result, most antibacterial drugs that are efficacious against gram-positive bacteria are ineffective against gram-negative bacteria. So, to reduce the misuse of antibiotics, new antibacterial medicines are being developed.^11,12^ Nanomaterials (1–100 nm) have so emerged as a viable alternative tool for combating multidrug-resistant bacteria. Nanomaterials’ physicochemical features provide a diverse platform for developing novel therapeutic techniques for multidrug-resistant bacteria.^13^ According to a recent study, ROS can generate oxidative stress in cells that damages bacterial cell organelles. Nanoparticles interact with the mercapto (−SH), amino (−NH), and carboxyl (−COOH) groups of proteins and nucleic acids, causing enzyme activity to be disrupted, cell structure to be altered, and the microorganism to be inhibited.^14−16^ Plants have been used to generate a variety of medicinal chemicals that could be used as biomaterials. *Ajuga parviflora* Benth. (Lamiaceae) is a well-known medicinal herb having antibacterial^17^ and antioxidant capabilities.^18,19^ The herb is used to cure liver disorders,^20^ dysentery, palsy, jaundice, arthritis, diabetes, and the tribal people also use it for calving (parturition).^21^

Zeolitic imidazolate framework-8 (ZIF-8) is a subclass of metal–organic framework (MOF) composed of zinc ions and 2-methylimidazole that crystallizes in a cubic lattice (space group *I*-43*m*) with a lattice constant of 16.10 Å (1.61 nm) and forms a sodalite topological crystal. Large molecules do not enter the pores since the pore-opening diameter of ZIF-8 is 3.4 Å (0.34 nm) and the pore-cavity diameter is 11.6 Å (1.16 nm). It exhibits excellent thermal stability up to 550 °C in an N_2_ atmosphere and no structural degradation in boiling organic solvents and water at 50 °C for 7 days.^22^ For gases like hydrogen and methane, ZIF-8 is increasingly gaining importance to be employed as a storage medium^23^ and as a high-capacity adsorbent to meet various separation needs^24^ in thin-film devices,^25^ catalysis,^26^ biomedical imaging,^27^ and drug delivery.^28^ Antibiotics such as vancomycin, doxorubicin (DOX),^29^ ciprofloxacin,^30^ gentamicin, and others have been encapsulated using ZIF-8.^31^ Cu^2+^-doped ZIF-8 loaded with curcumin was shown to alter its synergy with phenolic antioxidants, enhancing its antibacterial action.^32^

We are the first to encapsulate bioactive molecules from the *Ajuga parviflora* extract (APE) using the ZIF-8 architecture to create a nanocomposite (dubbed APE@ZIF-8). UV–vis absorption spectra, powder X-ray diffraction (PXRD), Brunauer–Emmett–Teller (BET), thermogravimetric analysis (TGA), scanning electron microscopy (SEM), high-resolution transmission electron microscopy (HRTEM), ζ-potential test, and dynamic light scattering are used to characterize ZIF-8 and APE@ZIF-8 NPs synthesized in the lab. The antibacterial activity of the APE@ZIF-8 nanocomposite is then tested against methicillin-resistant *S. aureus* (MRSA) and cephalosporin-carbapenem-resistant *E. coli* (CCREC). By comparing their zone of inhibition (ZOI) and minimum inhibitory concentration (MIC) values, it was discovered that APE@ZIF-8 NPs were more effective against bacteria than gentamicin, free ZIF-8, free APE, and methanol. In addition, future research into combination therapy is a possibility.

## Experimental Details

### Materials

#### Collection of Plant Materials

We took plant materials from Palampur, Himachal Pradesh, India. We identified plants for authentication and certification at the National Institute of Science Communication and Information Resources (Pusa Campus), New Delhi, India, and deposited a voucher specimen and a certificate to the Medicinal Plant Research Laboratory Department of Botany at Ramjas College, University of Delhi, New Delhi, India. I checked http://www.theplantlist.org and the database recognized the name *Ajuga parviflora* Benth. The record derives from WCSP (data supplied on 2012-03-23), which reports it as an accepted name (record 5361) with original publication details: 1: 59, *Pl. Asiat. Rar*., 1830 (see Figure S1b).

#### Chemicals

Zinc nitrate hexahydrate, 2-methylimidazole, Luria–Bertani (LB) agar, gentamicin, phosphate-buffered saline (PBS), and iodonitrotetrazolium chloride dye were from Sigma-Aldrich, and methanol was from Fisher Scientific.

#### Bacterial Culture

*S. aureus* MTCC-11941 (gram-positive) and *E. coli* MTCC-1652 (gram-negative) bacteria were collected from the Microbial Type Culture Collection, Chandigarh, India.

#### Soxhlation Method

To prepare the extraction process, we placed 25 g of shoot powder in a thimble filled with 150 mL of methanol. For 48 h, the extraction was carried out at 75 °C. The crude *A. parviflora* extract (APE) was filtered through Whatman filter paper grade 1 (thickness was 180 μm). The APE was retained at 75 °C until the solvent was completely evaporated using a rotary evaporator (see Figure S1d).

#### Gas Chromatography-Mass Spectrometry

Gas chromatography-mass spectrometry (GC-MS) analysis was carried out using a Shimadzu GC-MS-QP2010 Plus equipped with a programmable headspace autosampler and an autoinjector (see a detailed procedure in Figure S2).

#### Synthesis of ZIF-8

Two grams of zinc nitrate hexahydrate was dissolved in 20 mL of methanol; 4 g of 2-methylimidazole was dissolved in a separate flask containing 40 mL of methanol overnight under constant stirring at 400 rpm with a magnetic stirrer. The zinc nitrate hexahydrate solution was added to the 2-methylimidazole solution dropwise.

#### APE@ZIF-8 Synthesis

**Figure 1:**
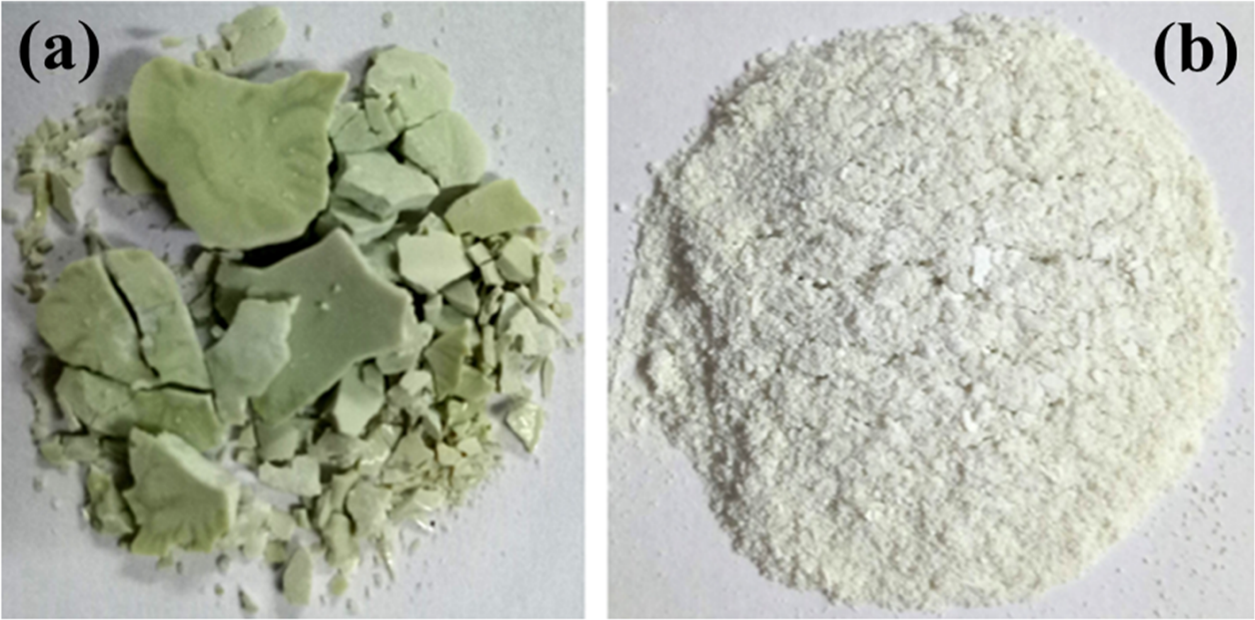
(a) APE@ZIF-8 nanocomposite flanks and (b) APE@ZIF-8 nanocomposite powder (photograph was taken using Lenovo Note K8) “Photograph courtesy of Ab Majeed Ahanger. Copyright 2021.”

Two grams of zinc nitrate hexahydrate was dissolved in 20 mL of methanol; 4 g of 2-methylimidazole and 1 g of APE were dissolved in 40 mL of methanol overnight with constant agitation at 400 rpm with a magnetic stirrer. Within an hour of stirring, the zinc nitrate solution was added dropwise into the solution of 2-methylimidazole containing APE, and the lime green reaction solution turned cream in color, due to the formation of the APE@ZIF-8 nanocomposite, and remained at rest for 24 h at room temperature. We centrifuged the solution at 10,000 rpm for 15 min to obtain the APE@ZIF-8 precipitate and then washed it three times with methanol (5 mL) to remove unreactive reagents before drying it at 70 °C. The APE@ZIF-8 nanocomposite flanks (Figure 1a) were ground into a powder using a mortar and pestle shown in Figure 1b.

### Antimicrobial Activity

#### Zone of Inhibition Assay

*S. aureus* and *E. coli* bacterial cultures were used as model organisms. We cultured bacteria in Luria–Bertani (LB) medium in a shaking incubator (110 rpm) at 37 °C for 24 h. Centrifugation collected the inocula at 10,000 rpm for 10 min. Finally, the density of bacteria was reduced to 10^5^ CFU mL^–1^. A 20 μL inocula pipette was drawn from the bacterial suspension of *S. aureus* and *E. coli* and spread all over the plate with a cotton swab. Different concentrations of ZIF-8, APE@ZIF-8, APE, gentamicin, and methanol were applied to sterile filter paper disks (6 mm in diameter) (thickness of 180 μm). The disks were firmly set on LB agar medium and inverted for incubation overnight.

#### Minimum Inhibitory Concentration Assay

The minimum inhibitory concentration (MIC) is the minimum concentration under which a compound’s antibacterial efficiency is determined. In this study, APE@ZIF-8 nanoparticles elicited the lowest concentration when compared to gentamicin, ZIF-8, APE, and methanol in both *S. aureus* and *E. coli*. Experiments were carried out in 96-well microtitre plates. The nutrient broth was added to each well, followed by the sample in varying concentrations, and finally, 20 μL of fresh bacterial culture (OD_600_: 0.4–0.5) was added. Using nutrient broth, we increased the last volume of each well to 200 μL. After an overnight incubation on a shaker incubator set to 37 °C and 120 rpm. Using an ELISA plate reader, we measured absorbance. Then, 30 μL of iodonitrotetrazolium chloride dye (INT dye) solution (0.25 mg/mL) prepared in autoclaved MilliQ water was added to each well, followed by incubation for 30 min at 37 °C. To assess growth and inhibition in treated and control wells, colorimetric visualization was used. Uncolored wells represented the inhibition of bacteria by a specific concentration of the test sample. The experiment was carried out in triplicate.

### Characterization Techniques

The chemical compounds are identified using gas chromatography-mass spectrometry analysis (GC-MS). To identify the different functional groups, the Fourier transform infrared spectroscopy (FTIR) absorption spectra were compiled from 4000 to 400 cm^–1^ with a scan speed of 2 cm^–1^. Ten milligrams of ZIF-8, APE, and APE@ZIF-8 were suspended in 1 mL of methanol for UV–vis absorption spectra, and a spectrum between 200 and 800 nm was recorded using a DLAB SP-UV1000 spectrophotometer. The samples were collected from the copper target Cu Kα radiation at 0.020° steps in 1.2 s in a 2θ range of 5–55° for powder X-ray diffraction (PXRD). A JEOL JSM 6610 scanning electron microscope (SEM) was used for the investigation. The powder was coated with platinum and viewed under the scanning electron microscope after being mounted on SEM stubs with double-sided sticky tape. To characterize the elementary composition, energy-dispersive X-ray spectroscopy was used in conjunction with scanning electron microscopy. A high-resolution transmission electron microscope (HRTEM) was used to determine the morphology and size of the synthesized material. To reduce primary particle agglomeration, the solution was sonicated for 30 min with ultrasound waves at a frequency of 20 kHz (20,000 cycles/s). Ten microliters of colloidal solution was dropped onto a 300-mesh copper grid covered with an ultrathin continuous carbon film, dried overnight in a desiccator, and examined under a transmission electron microscope. A surface area and pore size analyzer (BET) was used to measure the surface area and other parameters of ZIF-8 and APE@ZIF-8. Thermogravimetric analysis (TGA) was performed concurrently. We placed ∼15 mg of the sample in 200 μL open α-alumina crucibles and collected a thermogram from 25 to 900 °C at a heating rate of 10 °C/min. To determine the particle size distribution and electric charge of ZIF-8 and APE@ZIF-8 NPs, dynamic light scattering (DLS) measurements were performed three times, each time recording 20 measurements, and ζ-potential testing was used to confirm the surface charge of NPs.

## Results and Discussion

### Synthesis of APE@ZIF-8

Following previous methods in the literature,^32^ we successfully encapsulate APE into ZIF-8 framework. Metal ions and target organic molecules self-assemble to form a coordination polymer. Organic linkers are added to disassemble the metal ions from the target organic molecules and subsequently form ZIF-8 by the assembly of the metal ions and linkers. We encapsulated the target molecules during the formation of ZIF-8, resulting in hierarchical APE@ZIF-8. Several molecules with different functional groups have been successfully encapsulated into ZIF-8 crystals.

To find out chemical compounds responsible for antibacterial action, we subjected methanolic extraction of APE to GC-MS analysis and identified by comparing their retention times and mass weights with authentic samples by GC and the mass spectra from the Wiley Libraries, National Institute of Standards and Technology (NIST), and PubChem databases.^33^ The chemical compounds are listed in Table 1 along with their molecular formula and molecular weight. For the chromatogram of APE, see Figure S2.

**Table 1:** Chemical Components in APE, as well as Their Molecular Formula and Molecular Weight, Exhibit Antibacterial Characteristics

chemical compound	molecular Formula	molecular weight (g mol^–1^)	antimicrobial properties
stellasterol	C_28_H_46_O	398.70	antibacterial^34^
bruceantin	C_28_H_36_O_11_	548.60	anticancer^35−39^
squalene	C_35_H_62_O_7_	594.86	antitumor^40^
phthalimide	C_8_H_5_NO_2_	147.13	antimicrobial^41^ and anti-plasmodial^42^
oleic acid	C_18_H_34_O_2_	282.46	acaricide, herbicide, and insecticide^43^
bromoxynil heptanoate	C_11_H_22_O_2_	389.08	herbicide^44^
methyl palmitate	C_17_H_34_O_2_	270.50	acaricide^45^
phytol acetate	C_22_H_42_O_2_	338.60	antifungal^46^ and antibacterial^47^
octamethyltrisiloxane	C_8_H_24_O_2_Si_3_	236.53	antiparasitic and insecticide^48^
ethyl oleate	C_20_H_38_O_2_	310.50	acaricide^49^
phthalic acid	C_16_H_22_O_4_	278.34	disinfectant^50^
stigmasterol	C_29_H_48_O	412.70	anticonvulsant^51^

**Figure 2:**
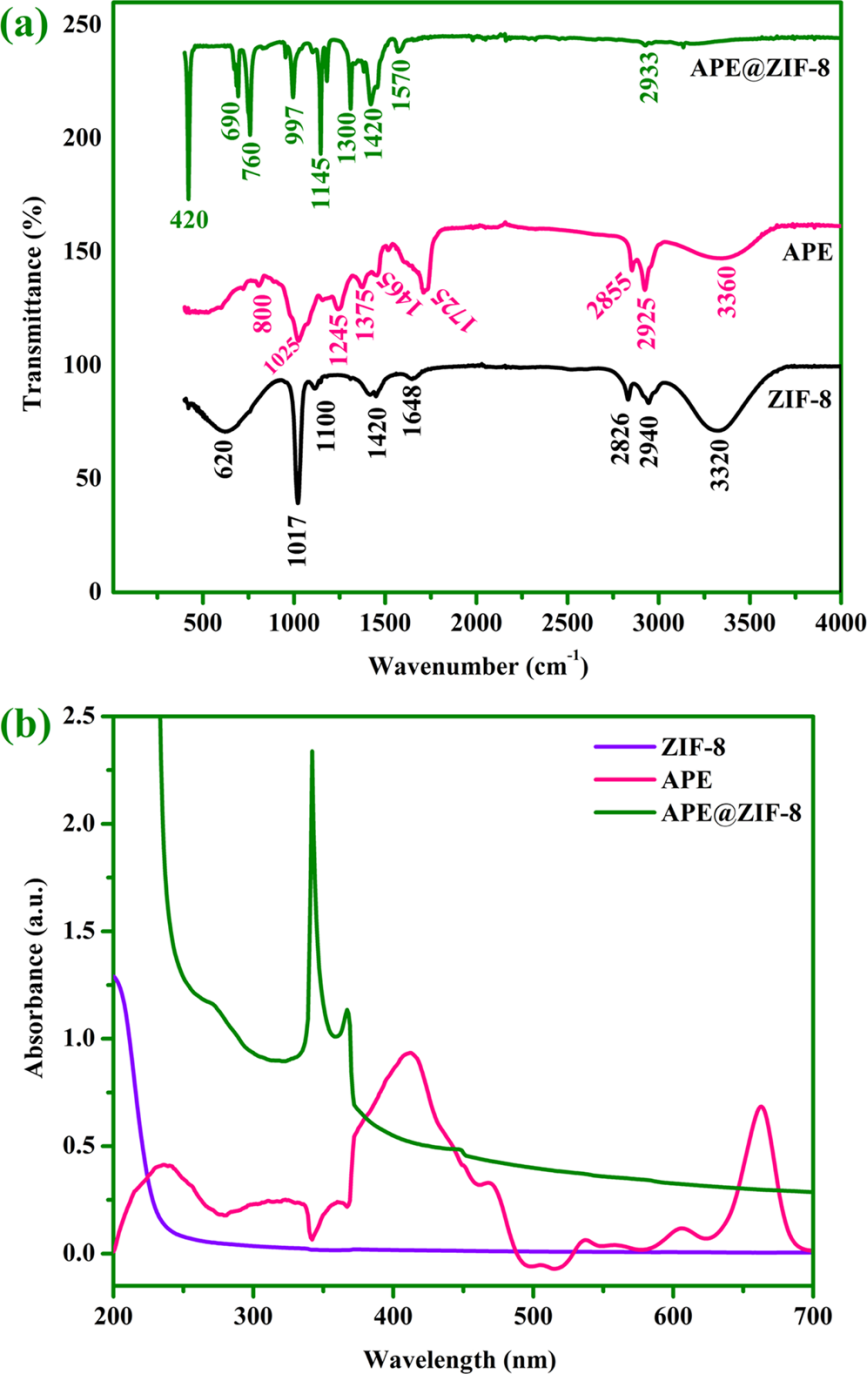
(a) Fourier transform infrared spectroscopy and (b) UV–vis absorption spectrum of ZIF-8, APE, and APE@ZIF-8 nanocomposite.

Further, Fourier transform infrared spectroscopy (FTIR) confirmed functional groups within the APE@ZIF-8 nanocomposite, APE, and ZIF-8 (examine Figure 2a). In ZIF-8, an absorption peak at 3320 cm^–1^ showed hydroxyl (OH) groups, and in APE, a sharp peak at 3360 cm^–1^ showed both amino (NH_2_) and hydroxyl groups. The positions and number of FTIR peaks for free ZIF-8 and free APE initially appeared to be quite close, implying that the types of chemical groups were similar. The peak at a stretching frequency of 2933 cm^–1^ was shown to have CH, CH_2_, and CH_3_ groups, which caused the C–H bond to stretch in APE@ZIF-8. Aliphatic compounds (methyl C–H asymmetric stretch) APE@ZIF-8 are visible in a narrow band between 2970 and 2950 cm^–1^. The peak at 1570 cm^–1^ confirms the aromatic group ring stretch (C=C–C) of APE@ZIF-8. A narrow band at 1420 cm^–1^ showing viny C–H in plan bend is present in APE@ZIF-8. Absorption bands at 997 cm^–1^ find vinyl terminals (−CH=CH_2_) in APE@ZIF-8. Aromatic C–H stretch is present at 760 cm^–1^ and is C–H 1,2-disubstitution (ortho). The aromatic ring (aryl) has a C–H monosubstitution (phenyl) between 690 and 760 cm^–1^. In the spectrum of APE@ZIF-8, the peak at 420 cm^–1^ was attributed to the Zn–N stretching.

We confirmed coordinated APE@ZIF-8 with Zn^2+^ ions by examining the UV–vis spectrum and comparing it to the ZIF-8 and APE spectra (examine Figure 2b). There was no discernible absorption peak for ZIF-8. APE exhibited a peak at 660 nm, which corresponded to chlorophyll a. When APE was incorporated into ZIF-8, APE@ZIF-8 had two major absorption peaks, one at 340 nm and the other at 360 nm. Intermolecular hydrogen bonds between the phenolic hydroxyl group in APE and the nitrogen atoms in 2-methylimidazole caused this encapsulation.^52^

**Figure 3:**
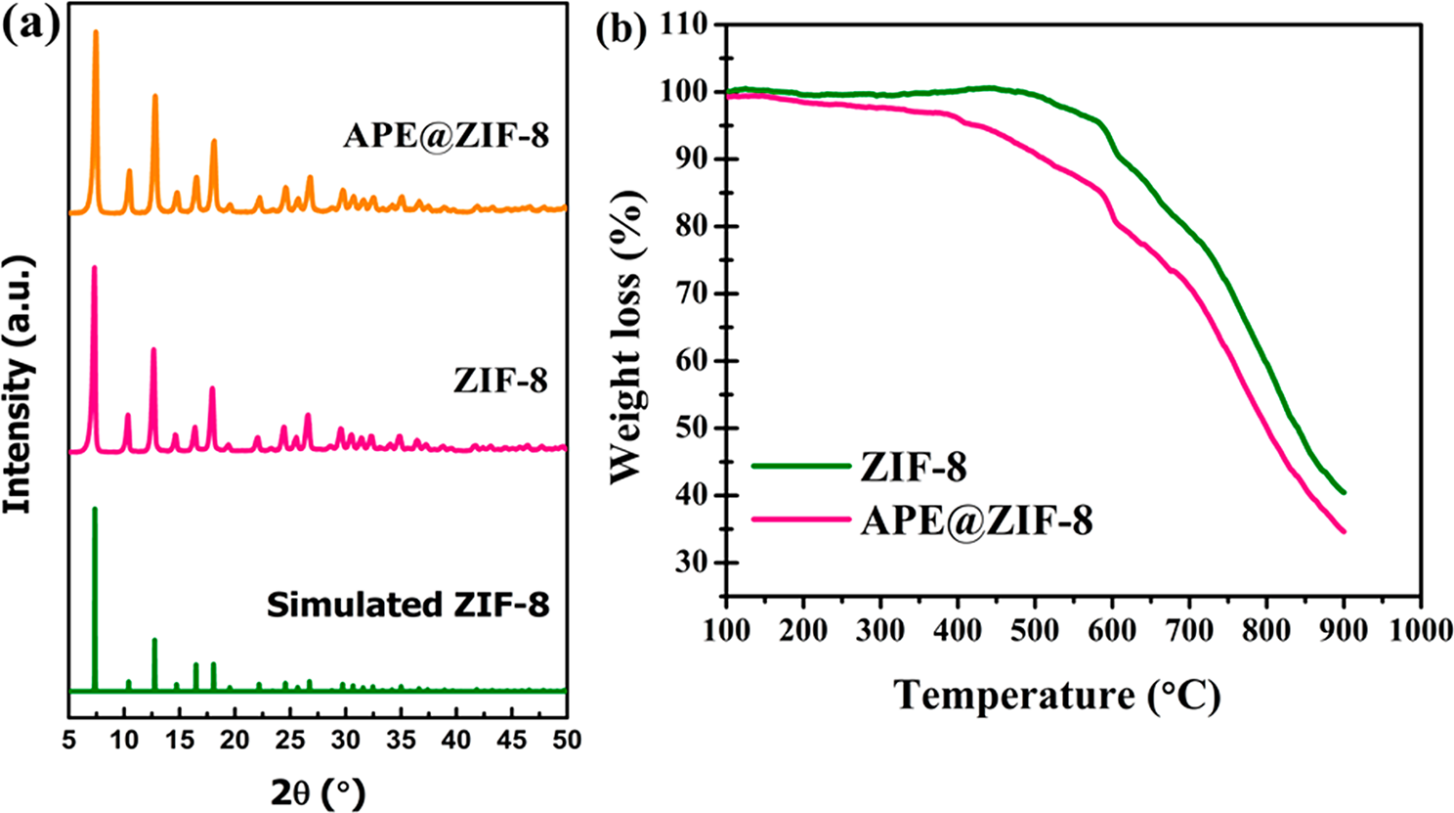
(a) Single-crystal PXRD pattern of APE@ZIF-8, ZIF-8, and simulated ZIF-8. (Crystallographic data of simulated ZIF-8 are available on the CCDC website under deposition number 602542.) (b) ZIF-8 and APE@ZIF-8 TGA thermograms.

PXRD analysis revealed that APE@ZIF-8 particles had high crystallinity. The broadening of the APE@ZIF-8 peak is due to APE in the pores/cavities of ZIF-8 crystals (examine Figure 3a). Peaks of ZIF-8 and APE@ZIF-8 agree well with those of simulated ZIF-8 and the cubic unit cell (JCPDS 00-062-1030 confirms it; *a* = *b* = *c* = 17.0116 Å and α = β = γ = 90°). The characteristic diffraction peaks of ZIF-8 and APE@ZIF-8 at 2θ = 7.3, 10.36, 12.66, 14.68, 16.34, 18.0, 19.42, 22.06, 24.5°, and 25.6, 26.64, 29.64, 30.52, 31.48, 32.38, 34.8°, which correspond to the planes of (011), (002), (112), (022), (013), (222), (114), (233), (134), and (044), respectively, are in compliance with previous articles.^53−57^

**Figure 4:**
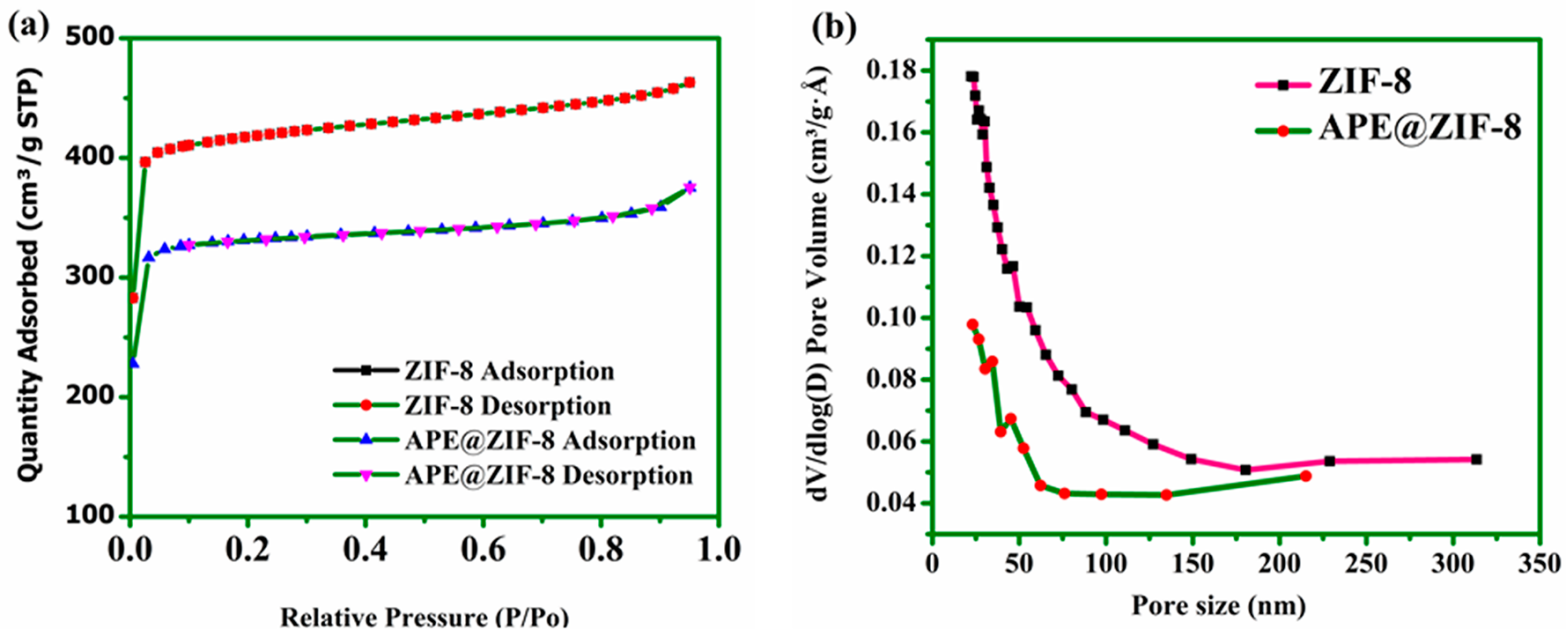
(a) Nitrogen sorption (adsorption–desorption) isotherms of ZIF-8 and APE@ZIF-8 nanoparticles and (b) pore size distribution of ZIF-8 and APE@ZIF-8 nanoparticles.

ZIF-8 and APE@ZIF-8 show a type I(b) isotherm with significant increases in N_2_ uptake at very low relative pressures, i.e., *P*/*P*_0_ < 0.12 and 0.0082, respectively. Following the abrupt initial increase in N_2_ adsorption, a perfect saturation in terms of *P*/*P*_0_ occurred.^58^ These characteristics are characterized by a high degree of microporosity in ZIF-8 and APE@ZIF-8. The porosity of our synthesized material is compared to that of commercially available ZIF-8, which has a BET surface area of >1300 m^2^/g.^59^ The gravimetric Brunauer–Emmett–Teller (BET) surface area of the APE@ZIF-8 nanocomposite is 963.73 m^2^/g, which is lower than our synthesized ZIF-8 (1317.27 m^2^/g) but higher than commercial ZIF-8 (>1300 m^2^/g). A decrease in the BET surface area of APE@ZIF-8 confirms that APE is encapsulated into framework of ZIF-8 (examine Figure 4a). ZIF-8 has a pore volume of 0.58 cm^3^/g, while APE@ZIF-8 has a pore volume of 0.44 cm^3^/g (see Figure S3). ZIF-8 has a pore width (size) of 2.17 nm, while APE@ZIF-8 has a pore width (size) of 2.15 nm (examine Figure 4b). The encapsulation of APE into ZIF-8 causes a decrease in the porosity of APE@ZIF-8. The parameters of the BET surface area are summarized in Table S1.

The TGA of ZIF-8 and APE@ZIF-8 under nitrogen flow (examine Figure 3b) reveals long buttes in the temperature range of 25–900 °C for both ZIF-8 and APE@ZIF-8. ZIF-8 can withstand temperatures of up to 500 °C. ZIF-8 has no weight loss up to 500 °C and a steep weight loss after 500 °C. The ZIF-8 curve showed a 5% weight loss at temperatures ranging from 500 to 550 °C. A weight loss of 20% occurred up to 650 °C, which was attributed to the decomposition of ZIF-8 as the framework structure collapsed. A loss of 35% occurred at 800 °C. Decomposition of 2-methylimidazole was observed between 800 and 900 °C, with an additional weight loss of 40% due to crystal structure collapse. The final weight loss implies that ZIF-8 has been completely converted to zinc oxide in an oxidative environment.^60^ We observed no weight loss with APE@ZIF-8 up to 400 °C. The TGA curve of APE@ZIF-8 shows a gradual weight loss from 400 °C onward, corresponding to the removal of APE. Because of the loss of water molecules, the TGA curve obtained for APE@ZIF-8 showed a slight weight loss of 5% between 400 and 450 °C. Because of the decomposition of organic molecules from APE@ZIF-8, we observed a weight loss of 10% between 450 and 650 °C. Furthermore, a weight loss of 20% was observed between 650 and 700 °C. Between 700 and 900 °C, a weight loss of 40% and decomposition of 2-methylimidazole were observed due to the collapse/disintegration of the crystal structure caused by partial degradation of ZIF-8 intermolecular bonds. These progressive weight losses validated the successful incorporation of APE into ZIF-8.

**Figure 5:**
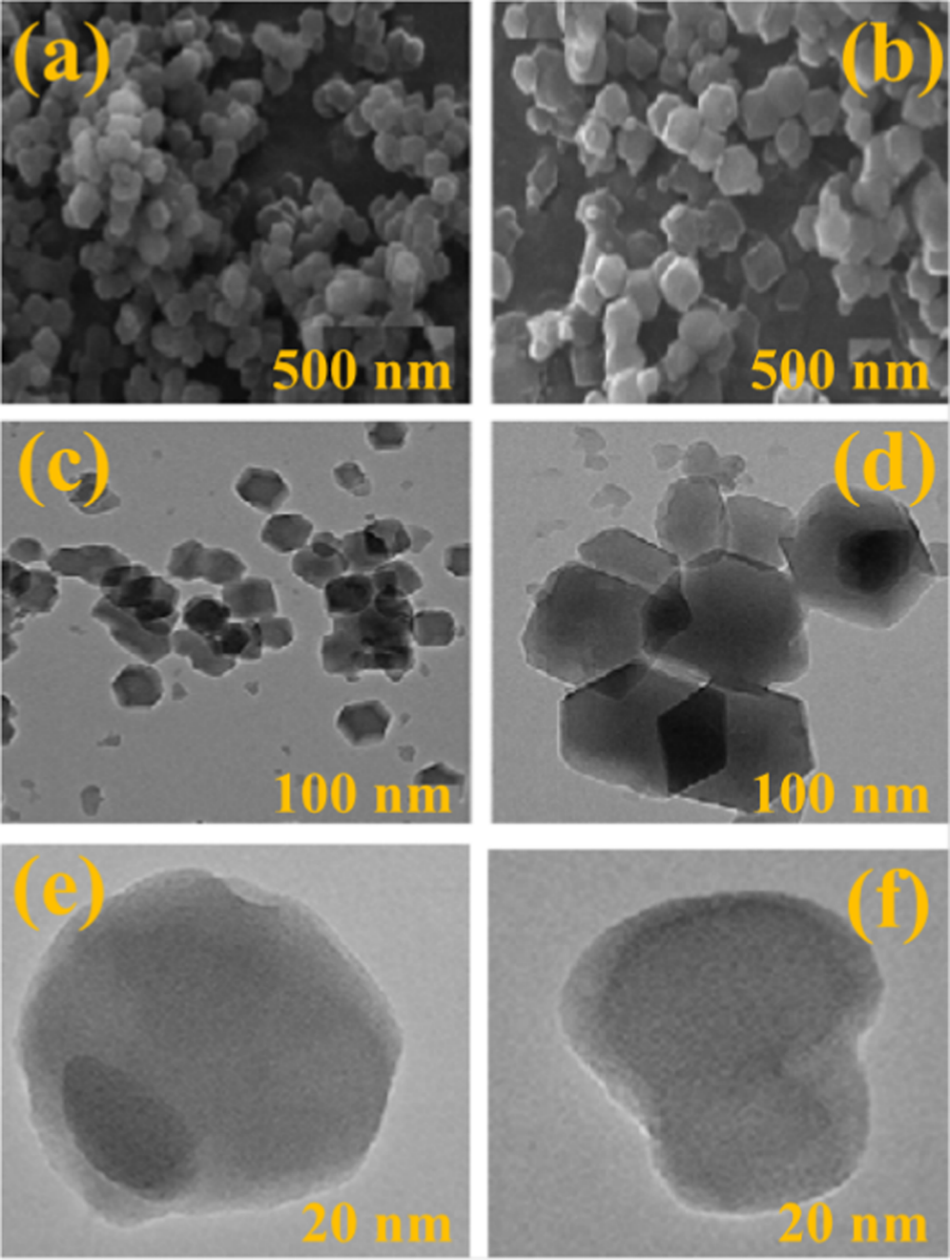
Magnified SEM monographs of (a) ZIF-8 and (b) APE@ZIF-8; TEM monographs of (c) ZIF-8 and (d) APE@ZIF-8; and magnified TEM monographs of (e, f) APE@ZIF-8.

According to the SEM and HRTEM monographs, the morphology of APE@ZIF-8 NPs is similar to that of pure ZIF-8 NPs. SEM images revealed that ZIF-8 (see Figure 5a) and APE@ZIF-8 (see Figure 5b) have truncated rhombic dodecahedron morphologies with smooth surfaces, which is consistent with previously reported ZIF-8 crystal shapes,^61,62^ and the average particle size is 0.5 μm. The TEM study was carried out to investigate detailed microstructures and the actual particle size of synthesized materials. According to TEM monographs, the morphology of APE@ZIF-8 NPs (see Figure 5d) is consistent with ZIF-8 (see Figure 5c), and there is no significant difference between ZIF-8 and APE@ZIF-8 NPs. The size is dispersed and ranges from 20 to 100 nm. The ZIF-8 and AP@ZIF-8 NPs were discovered to contain Zn, O, N, and C by energy-dispersive X-ray spectroscopy (see Figure S4a,b).

**Figure 6:**
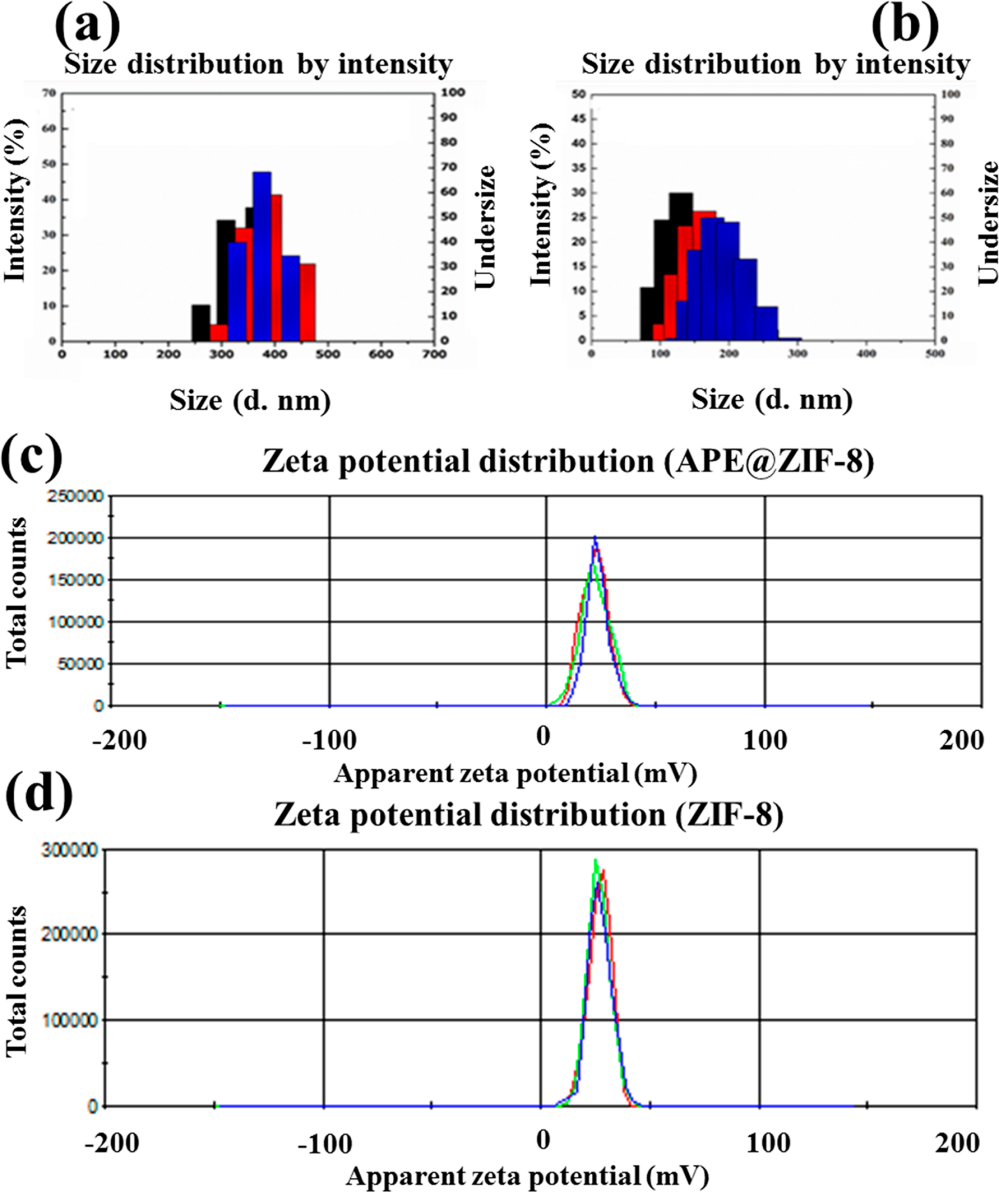
Hydrodynamic size distribution results of APE@ZIF-8 (a) and ZIF-8 (b). ζ-potential distributions of APE@ZIF-8 (c) and ZIF-8 (d).

We performed DLS analysis to determine the hydrodynamic particle size to supplement the TEM results. We performed three measurements to determine the size and stability of nanoparticles, and each measurement was recorded 20 times to study the particle size distribution of nanoparticles. As shown in Figure 6a,b, the average particle size of APE@ZIF-8 NPs was 237.9 nm with a polydispersity index (PDI) of 0.247, which is slightly larger than that of ZIF-8 NPs (236.4 nm with a PDI of 0.285). The hydrodynamic diameter showed no size change, indicating that the APE@ZIF-8 nanocomposite is stable. APE@ZIF-8 has a ζ-potential of +25.5 mV, while ZIF-8 has a ζ-potential of +27.3 mV (examine Figure 6c,d). ZIF-8 has a high positive charge (+27.3 mV) but lacks bactericidal APE. Only APE@ZIF-8 exhibited superior antibacterial properties. It carried strong local surface positive charges (+25.5 mV) to lower *S. aureus* and *E. coli* membrane potentials. Furthermore, because APE was encapsulated within the cavities and pores of ZIF-8, its stability and solubility were significantly improved.

**Figure 7:**
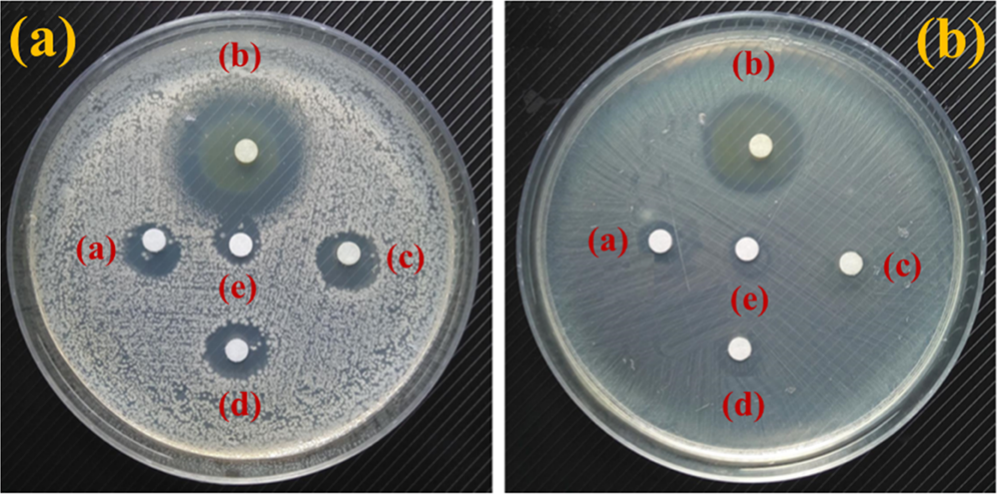
Typical disk diffusion pictures of ZOI in (a) *S. aureus* and (b) *E. coli* after treatment ((a) = ZIF-8, (b) = APE@ZIF-8, (c) = APE, (d) = gentamicin, and (e) = methanol). (The photograph was taken using Lenovo K8.) “Photograph courtesy of Ab Majeed Ahanger. Copyright 2021.”

**Figure 8:**
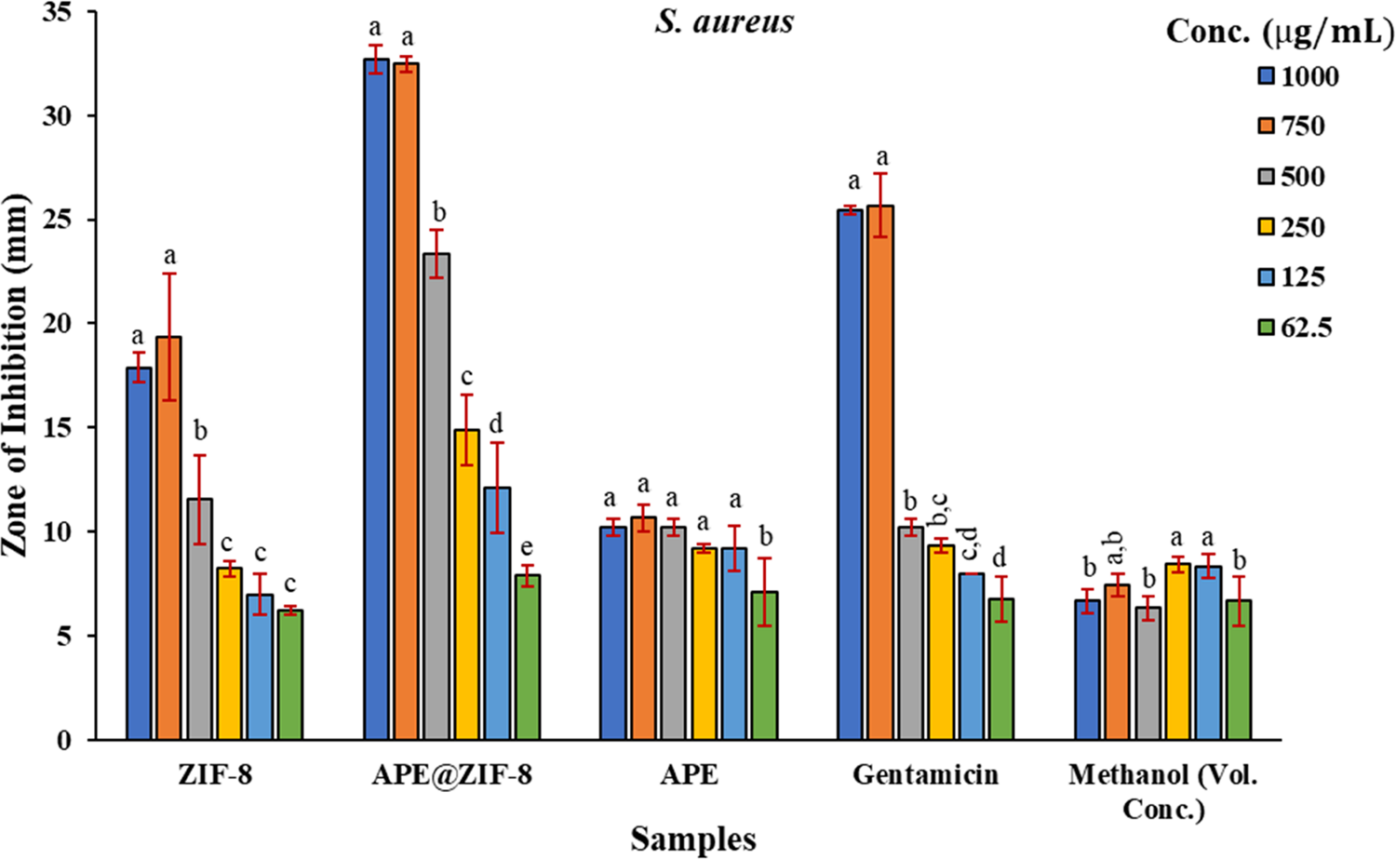
Zone of inhibition of ZIF-8, APE@ZIF-8, APE, gentamicin, and methanol in *S. aureus* shown on the histogram. Each statistic is the average of three biological replicates ± SD. According to Duncan’s LSD test, values that share common lower case letters are insignificant at *P* ≤ 0.05.

**Figure 9:**
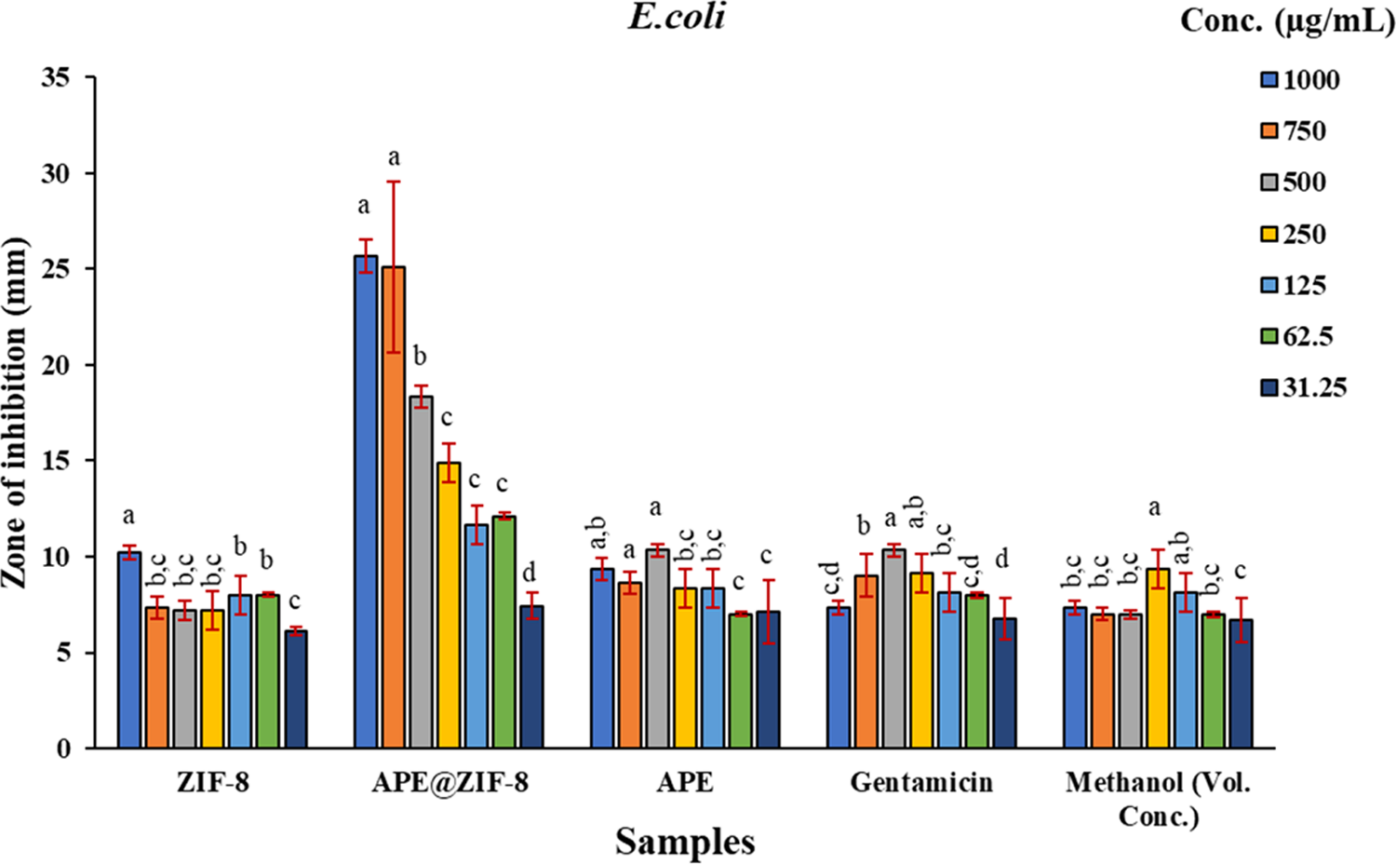
Zone of inhibition of ZIF-8, APE@ZIF-8, APE, gentamicin, and methanol in *E. coli* shown on the histogram. Each statistic is the average of three biological replicates ± SD. According to Duncan’s LSD test, values that share common lower case letters are insignificant at *P* ≤ 0.05.

The antibacterial effectiveness of the APE@ZIF-8 nanocomposite compared to the control group was evaluated using different concentrations of ZIF-8, APE, methanol, and standard (gentamicin) at 1000, 750, 500, 250, 125, 62.5, and 31.25 μg mL^−1^(methanol in volume concentration) against *S. aureus* (gram-positive) and *E. coli* (gram-negative) bacteria. In all concentrations, APE@ZIF-8 NPs had the highest zone of inhibition (ZOI) in both bacterial species, followed by gentamicin, ZIF-8, APE, and methanol (examine Figure 7a,b). The histograms (see Figures 8 and 9) show ZOI values for ZIF-8, APE@ZIF-8, APE, gentamicin, and methanol, demonstrating different antimicrobial activities against the tested strains. Zone of inhibition (ZOI) obtained after incubating bacteria with APE@ZIF-8 NPs is very high, indicating a positive response to gentamicin. Antibacterial activity of ZIF-8 and APE was not promising, but it increased in conjugation. Bacterial growth decreases as APE@ZIF-8 concentration increases. Because of the differences in the cell wall structure, APE@ZIF-8 had significantly different bactericidal activities against *S. aureus* and *E. coli*. *S. aureus* cells are spherical (0.5–1.5 μm in diameter). The two main components of the *Staphylococcus* cell wall are peptidoglycan and teichoic acid. *E. coli* has an average diameter of 2.0–0.5 μm.^63^ The approximate pore size of a bacterial cell ranges from 5 to 50 nm,^64^ and the size of APE@ZIF-8 NPs ranges from 20 to 100 nm (see Figure 5e,f). As a result, APE@ZIF-8 NPs are expected to attach to and penetrate the bacterial cell membrane via the electrostatic attraction of their positively charged surfaces and negatively charged bacterial surfaces, causing cell wall damage. The APE@ZIF-8 NPs pierced the bacterial cell wall of *S. aureus* and *E. coli* and leaked out of intercellular components, most likely causing cell lysis and interfering with the translocation process in the formation of tRNA to inhibit protein synthesis. The displacement of cations causes cell wall rupture, which aids in the linking of lipopolysaccharide (a bacterial endotoxin) to others. ROS production causes oxidative stress in cells, destroying the cell membrane, DNA, and protein. This mechanism kills the bacteria at an early stage, resulting in cell death. The findings show that APE@ZIF-8 can function as an effective nanobacterial agent.

**Figure 10:**
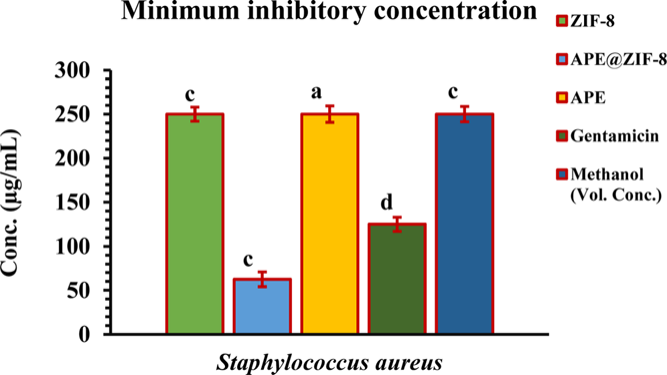
MICs of ZIF-8, APE@ZIF-8, APE, gentamicin, and methanol against *S. aureus* shown in the histogram.

**Figure 11:**
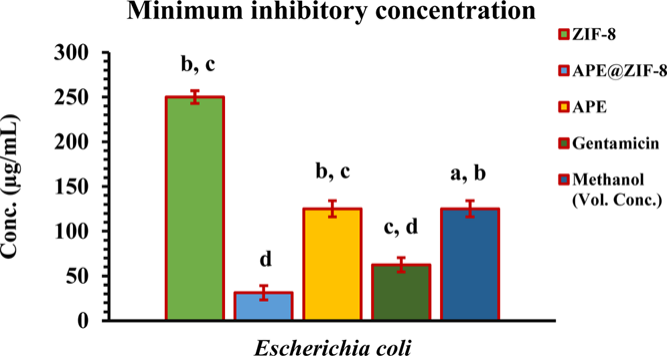
MICs of ZIF-8, APE@ZIF-8, APE, gentamicin, and methanol against *E. coli* shown in the histogram.

Minimum inhibitory concentration (MIC) is noted where no visible growth is found in 96-well microtitre plates after treatment. MIC values of APE@ZIF-8 NPs were found to be 62.5 μg/mL for *S. aureus* and 31.25 μg/mL for *E.coli* (examine Figures 10 and 11). APE@ZIF-8 NPs elicited their least concentration when compared to ZIF-8, APE, gentamicin, and methanol against methicillin-resistant *S. aureus* (MRSA) and cephalosporin-carbapenem resistant *E. coli* (CCREC) (examine Figures 8 and 9). The MIC of APE@ZIF-8 is also double that of gentamicin, indicating that APE@ZIF-8 NPs interacted well with the cellular structure of bacteria, disrupting cellular integrity and, ultimately, inhibiting growth. The results showed that using APE@ZIF-8 against antibiotic-resistant bacteria (*S. aureus* and *E. coli*) would reduce the antibiotic load and further decrease the resistance created in the bacteria as a result of excessive and unusual antibiotic use.

### Statistical Analysis

The mean of the ZOI for each combination in each experiment was calculated using MS Excel 2019 and shown as the mean ± SD of three replicates in the preliminary investigation of the role of two bacterial strains and diverse samples in their zone of inhibition. To examine the significant (*P* ≤ 0.05) differences between different concentrations, a one-way analysis of variance (ANOVA) with Duncan’s tests was used with SPSS software (IBM SPSS Statistics version 23). The antibacterial activities of ZIF-8, APE@ZIF-8, APE, gentamicin, and methanol against two bacterial strains were evaluated using basic statistics based on the presence of an inhibitory zone and determination of its diameter using the disk diffusion assay. ZIF-8 first demonstrated an increasing trend in ZOI when the concentration of ZIF-8 was reduced from 1000 to 750 μg mL^−1^ in *S. aureus*. Then, from 750 to 500 μg mL^−1^, ZOI decreased (*P* ≤ 0.05), and from 500 to 250 μg mL^−1^, ZOI decreased significantly (*P* ≤ 0.05). However, from 250 to 62.5 μg mL^−1^ ZOI showed a decreasing tendency, as shown in Figure 8. ZOI values were nearly the same with APE@ZIF-8, with a continuous lowering trend from 1000 to 750 μg mL^−1^ concentration. However, the concentration decreased from 500 to 62.5 μg mL^−1^, resulting in lower ZOI values (*P* ≤ 0.05). With APE, the ZOI values increased significantly (*P* ≤ 0.05) from 1000 to 125 μg mL^−1^, but at the lowest concentration of 62.5 μg mL^−1^, the ZOI value decreased dramatically (*P* ≤ 0.05) from 9.22 mm in 125 (*P* ≤ 0.05) to 7.11 mm in 62.5 (*P* ≤ 0.05). ZOI values were unaffected by the concentration of gentamicin, which ranged from 1000 to 750 μg mL^−1^ (25.44 and 25.66 mm, respectively). The concentrations decreased from 750 to 62.5 μg mL^−1^. ZOI values decreased (*P* ≤ 0.05), and the lowest zone of inhibition was found at 62.5 μg mL^−1^ (6.77 mm). The maximum ZOI (8.44 mm) was obtained at a concentration of 250 μg mL^−1^, followed by a decrease (8.33 mm) at 125 μg mL^−1^, and the lowest ZOI was 6.33 mm at 500 μg mL^−1^.

The ZIF-8 originally showed a declining trend in ZOI from 1000 to 750 μg mL^−1^ in *E. coli* (7.33 mm). Then, at 500 and 250 μg mL^−1^, the ZOI was the same (7.22 mm). However, the ZOI is higher (8.00 mm) at doses of 125 and 62.5 μg mL^−1^ than that at 500 and 250 μg mL^−1^. In terms of ZOI (6.11 mm), concentrations of 31.25, 500, and 250 μg mL^−1^ show a decreasing tendency, as shown in Figure 9. From 1000 to 125 μg mL^−1^, the ZOI values (*P* ≤ 0.05) decreased with APE@ZIF-8. When compared to greater concentrations, the ZOI (12.11 mm) of 62.5 μg mL^−1^ exhibited a modest increase (12.11 mm). The ZOI is lowest in the case of a concentration of 31.25 μg mL^−1^. The APE’s ZOI values decreased from 1000 to 750 μg mL^−1^, indicating a decreasing trend. Among all APE concentrations from 1000 to 31.25 μg mL^−1^, 500 μg mL^−1^ had the greatest ZOI value (10.33 mm). Gentamicin had a ZOI value of 7.33 mm at 1000 μg mL^−1^ and did not show a decreasing tendency from higher concentrations to lower concentrations, and concentrations of 750 and 250 μg mL^−1^ had almost the same ZOI values as a concentration of 500 μg mL^−1^, which had a greater ZOI value of 10.33 mm. The lowest zone of inhibition was found at a concentration of 31.25 μg mL^−1^, and the highest zone of inhibition was present at a dosage of 125 μg mL^−1^ (6.77 mm). With methanol, the first three concentrations had nearly identical ZOI values, ranging from 1000 to 500 μg mL^−1^. The ZOI values decreased in order from 250 to 31.25 μg mL^−1^.

## Conclusions

In conclusion, we created the nanoantibacterial agent (APE@ZIF-8) using a one-step procedure that encapsulated bioactive molecules from leaf extract of *A. parviflora* Benth. into ZIF-8. APE@ZIF-8 nanocomposite has excellent biocompatibility, stability, and drug release candidate carriers. Experiments show that APE@ZIF-8 NPs can stop methicillin-resistant *S. aureus* (MRSA) and cephalosporin-carbapenem-resistant *E. coli* (CCREC) bacteria from growing. The positively charged APE@ZIF-8 NPs attach with the negatively charged bacterial surface by electrostatic interactions, resulting in the production of reactive oxygen species (ROS) that damages cellular organelles and the genetic material of bacterial cell and finally causes cell death.

## Supporting Information Available

Collection of plant materials (Figure S1); gas chromatography-mass spectrometry analysis (GC-MS) analysis of the methanol extract of *A. parviflora* (Figure S2); pore volume of ZIF-8 and APE@ZIF-8 (Figure S3); EDX of (a) ZIF-8 and (b) APE@ZIF-8, collected using a JEOL scanning electron microscope (Figure S4); and various parameters of ZIF-8 and APE@ZIF-8 evaluated from BET analysis (Table S1) (PDF)
