# Supplementary material for: Synthesis and Encapsulation of Ajuga parviflora Extract with Zeolitic Imidazolate Framework-8 and Their Therapeutic Action against G+ and G– Drug-Resistant Bacteria
Source: ACS Omega. 2022 Jan 6;7(2):1671–81. doi: 10.1021/acsomega.1c03984 (PMC8772321; doi:10.1021/acsomega.1c03984)
Supplement: Supplementary file 1 — ao1c03984_si_001.pdf [file ao1c03984_si_001.pdf]

Supporting Information for Publication

Synthesis and Encapsulation of *Ajuga parviflora* Extract with Zeolitic Imidazolate Framework-8 and Their Therapeutic Action Against G+ and G- drug-resistant bacteria

††Ab Majeed Ahanger[1], Suresh Kumar*[1], Atul Arya[1], Amrita Suryavanshi[1], Dolly Kain[1], ‡‡Vandana[2]

[1]University of Delhi Faculty of Science, Department of Botany, Ramjas College, Medicinal Plant Research Laboratory

[2]University of Delhi Faculty of Science Department of Chemistry, Dyal Singh College

*Corresponding Author: Professor Suresh Kumar
Email Id: suresh.kumar@ramjas.du.ac.in
Mobile No: 011 9868210236

†† Department of Environmental Studies University of Delhi, New Delhi-110007 India.

‡‡ Medicinal Plant Research Laboratory, Department of Botany, Ramjas College, University of Delhi, New Delhi-110007 India

Collection of plant materials:

The plant materials for this study were collected in Palampur, Himachal Pradesh, India. Palampur is in India's Kangra district, in the Northwestern Himalayan biogeographic zone, between 32.12 N latitude and 76.53 E longitude. The plants were collected and dried in a plant press so that they could be identified (see Figure 1b). The plant specimen was identified and authenticated with (Reference No NISCAIR/RHMD/Consult/2020/ 3083-32) at the Council of Scientific and Industrial Research-National Institute of Science Communication and Information Resources (CSIR-NISCAIR) Vigyan Sanchar Bhawan, Dr. K.S. Krishnan Marg, Pusa Campus, New Delhi, India. The plants were rinsed in distilled water 2-3 times. Before drying, the roots and shoots were separated. The leaves were dried in the shade at ambient temperature (22°C) to allow for evaporation. With the use of a mortar and pestle, the crisp/brittle leaves were ground into a fine powder (see Figure S1c), which was then stored in an airtight plastic container with a label for future use.

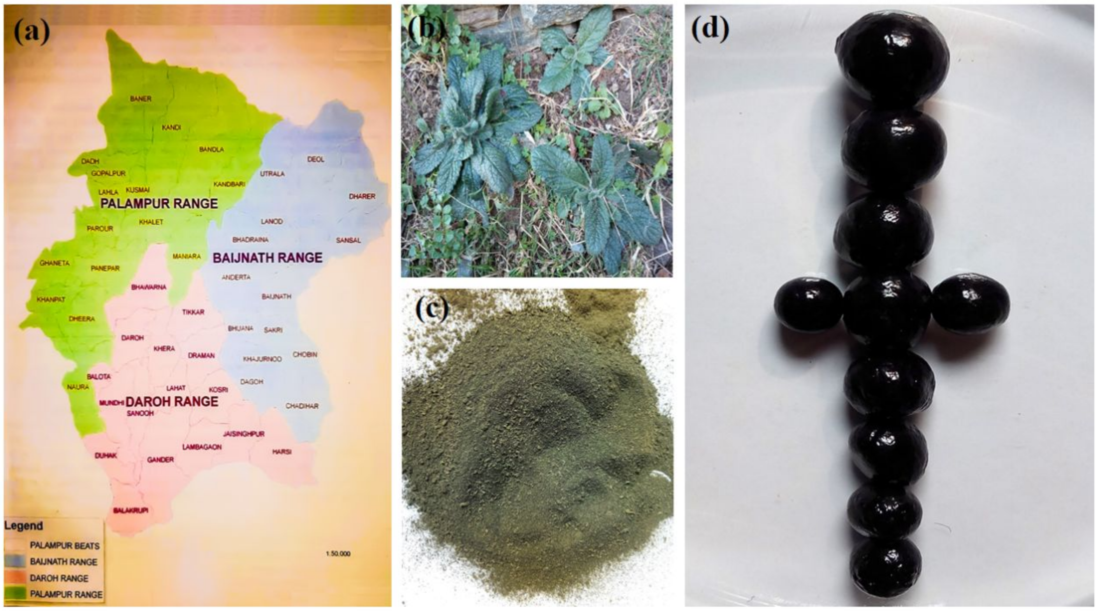

Figure S1. (a) Plant collecting location (b) *Ajuga parviflora* Benth. the wild herb that grows in the hills of Palampur, Himachal Pradesh, India. (c) leaf powder (d) *Ajuga parviflora* extract pellets (Photographs b-d were captured@Lenovo Note K8) “Photograph courtesy of Ab Majeed Ahanger. Copyright 2021.

Gas chromatography-mass spectrometry analysis (GC-MS) analysis:

The DB-1/RTX-MS (30 meters) capillary column was utilized, with helium as the carrier gas and a flow rate of 3 mL/min and a 1L injection volume. The column was heated to 100°C for 2 minutes after injection, then increased to 170°C using a 10°C/min heating ramp for 8 minutes to analyze APE. The final temperature was then boosted to 240°C for 15 minutes using a 10°C/min heating ramp. At 250°C, the injections were done in split mode (30:1). The temperature of the detector and injector was set to 260°C and 250°C, respectively, the pressure was set to 76.2 kPa, and the sample was run for 70 minutes. For the flame ionization detector (FID), the temperature and nominal beginning flow were set to 230°C and 3.1 mL/min, respectively. The following were the MS parameters: The scan range (m/z) is 40-650 atomic mass units under electron impact (EI) ionization (70 eV) (AMU).

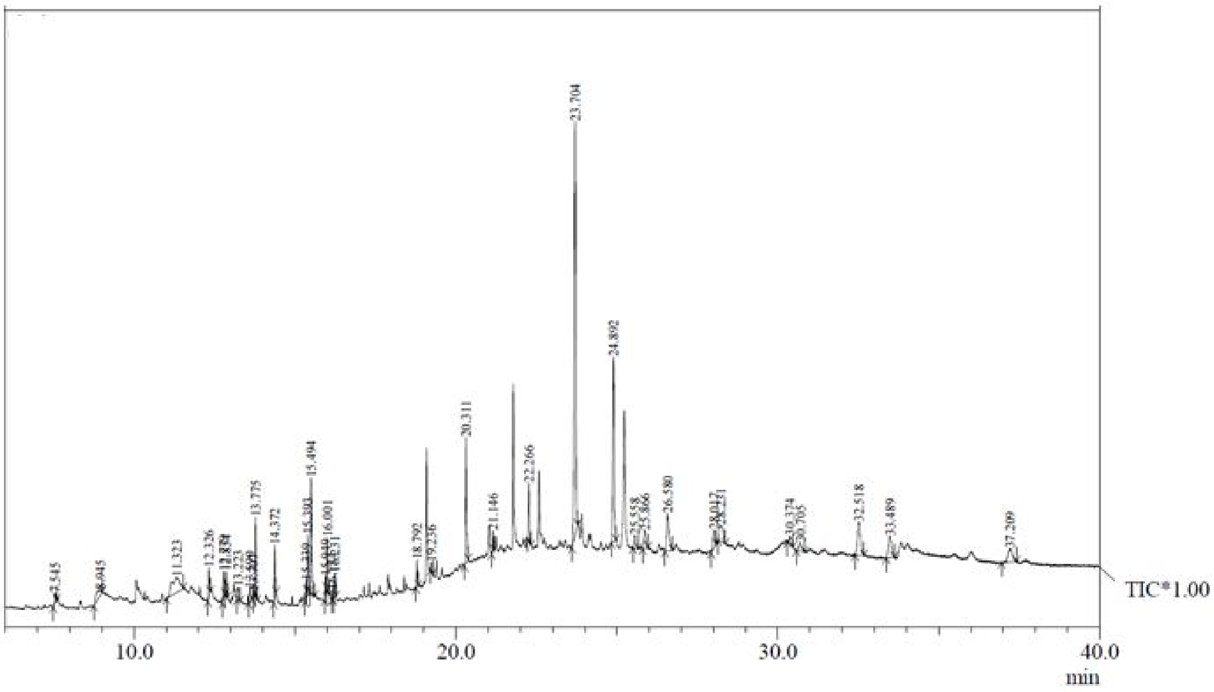

Figure S2. Chromatogram of *Ajuga parviflora* methanol extract was analyzed using gas chromatography-mass spectrometry (GC-MS)

The pore volume of ZIF-8 and APE@ZIF-8:

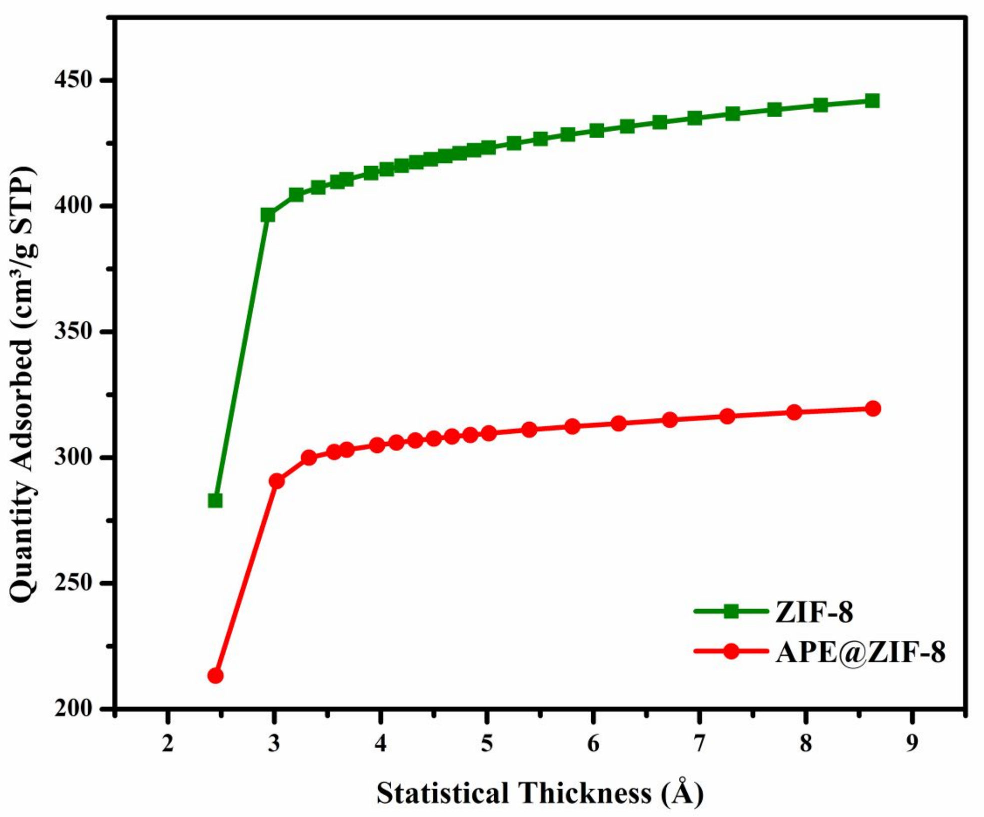

Figure S3. The pore volume of ZIF-8 and APE@ZIF-8

The Energy Dispersive X-Ray revealed that the ZIF-8 and AP@ZIF-8 NPs comprise Zn, O, N, and C

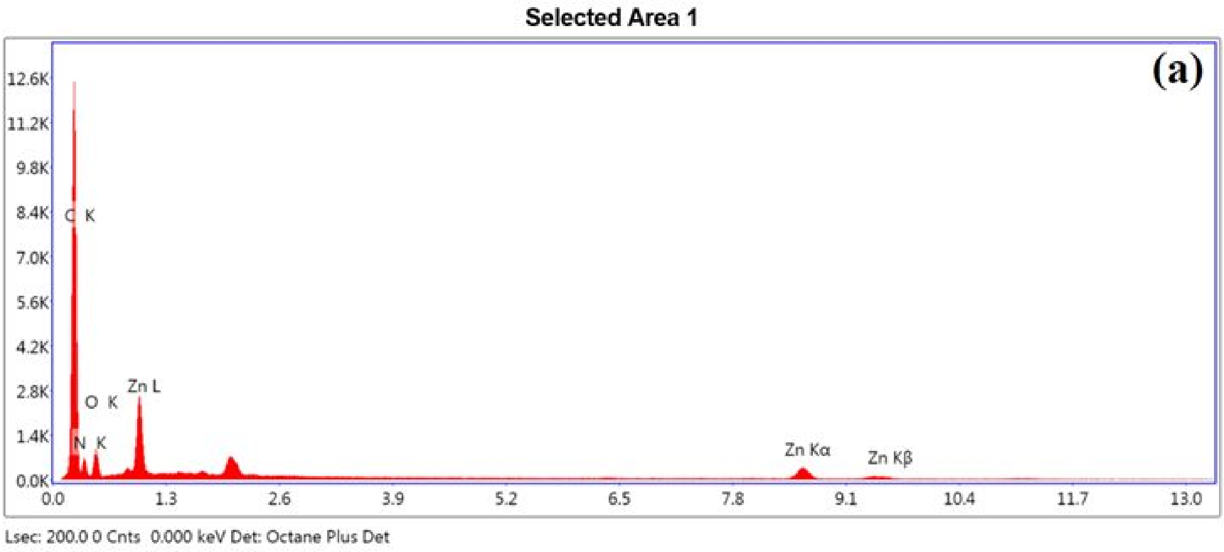

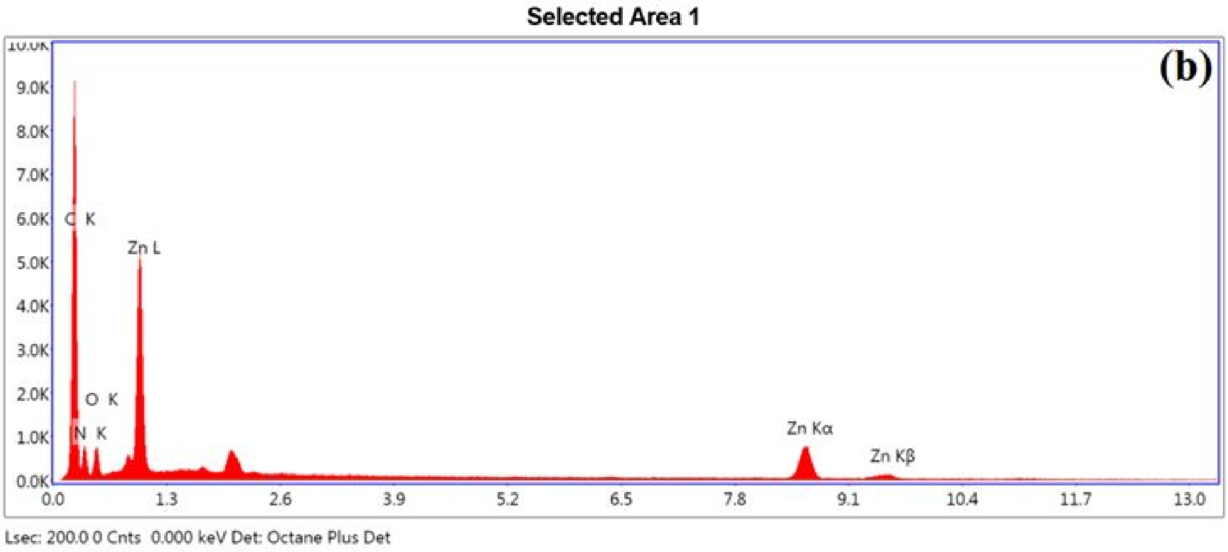

Figure S4. (a) EDX of ZIF-8 and (b) APE@ZIF-8 taken with a JEOL scanning electron microscope.

Table S1. Various parameters of ZIF-8 and APE@ZIF-8 were evaluated from BET analysis.

**Surface Area**	**ZIF-8**	**APE@ZIF-8**
Single point surface area at P/Po = 0.30:	1288.58 m^2/g	942.62 m^2/g
BET Surface Area:	1317.27 m^2/g	963.72 m^2/g
Langmuir Surface Area:	1852.88 m^2/g	1356.65 m^2/g
t-Plot Micropore Area:	1166.11 m^2/g	882.27 m^2/g
t-Plot External Surface Area:	151.15 m^2/g	81.45 m^2/g
**Pore Volume**		
t-Plot micropore volume:	0.57 cm^3/g	0.43 cm^3/g
**Pore Size**		
Adsorption average pore width (4V/A by BET):	2.17 (nm)	2.15 (nm)
BJH Adsorption average pore diameter (4V/A):	4.91 (nm)	5.60(nm)
BJH Desorption average pore diameter (4V/A):	4.89 (nm)	5.38 (nm)
